# Neurosis of the Stomach from a Surgical Standpoint

**Published:** 1898-01

**Authors:** B. E. Hadra

**Affiliations:** San Antonio, Texas


### TEXAS MEDICAL JOURNAL.

ESTABLISHED JULY, 1885.

PUBLISHED MONTHLY.—SUBSCRIPTION $2.00 A YEAR.

VOL. XIII. AUSTIN, JANUARY, 1898. No. 7.

### Original Contributions.

For the Texas Medical Journal.

### Neurosis of the Stomach From a Surgical Standpoint.

BY B. E. HADRA, M. D., SAN ANTONIO, TEXAS.

Partially read before the West Texas Medical Association at San Antonio, November 25, 1897.

Neurosis of the stomach is a term of convenience for the designation of such diseases as are not explainable by visible changes of the tissues of this organ. A neurosis then may be due to a disturbance of the stomach itself, not amenable to diagnosis; or to the influence of some disorder situated outside of the stomach. It is this latter class which I will review in my paper, and I intend to do it with special reference to surgery.

The manner in which a distant disturbance acts upon the stomach is three fold: (1) by toxic agents (through blood and lymph.) (2) by so-called nervous reflex—though a combination of both of these is not uncommon; and (3) by mechanical interference.

The first process is an everyday observation. We see the most varied disturbances of gastric secretions in all zymotic, we may say, in all feverish diseases. The most prominent, though, are those in which pus has formed and remains free or becomes encapsulated. It is then an auto-intoxication, and since the ingenious experiments of Hitzig and Alt have proven that a large percentage of the morphine injected hypodermically is in a very short time eliminated into the stomach, and that it then acts upon the nerve terminations within that organ so as to cause vomiting; and since a similar excretion of other poisonous or at least foreign substances by the stomach has been found to occur, the supposition is altogether reasonable that the toxins due to suppuration and similarly allied changes in the body tissues, in short, that some products of disordered katabolism should also pursue the same course of elimination through the stomach. It is surprising that practically we take so little notice of the fact that the stomach and small bowels are largely excretory and eliminative organs. They are mostly regarded as absorbing instruments. It is only necessary to observe the inexhaustible vomiting in a full-fledged case of peritonitis to understand to what extent the small intestines and stomach may eliminate effete material. Such a view explains the almost constant sympathy of the stomach with nearly every disorder of the system.

The second mechanism mentioned was that of nervous reflex. Not deeming it necessary to go into an elaborate explanation, I will only say that the stomach has its own ganglia within its walls (Auerbach's, Meissner's plexus), which are no doubt in connection with the sympathetic as well as with the pneumogastric and the cerebro-spinal nerves, so that every part of the body will have a chance to be heard from. This is another reason why the stomach may be regarded as a kind of central alarm station.

The third method of causing neurosis is by mechanical interference, either by pressing on the organ, or by pulling it unduly in an abnormal direction, or by interfering with its normal movements. In my opinion, Byron Robinson is perfectly correct in maintaining that this last cause is the most common and potent one as a pathological factor. It is easy to imagine that exactly as a sudden and forcible interruption of a strong muscular action or a sudden reversed action becomes extremely painful by producing a jolt, so also may a sudden interruption of a peristaltic action of the stomach or of an intestinal loop manifest itself as a most unpleasant sensation.

As to the symptoms of neurosis, I may say that almost every kind of disorder has to be included, sensory as well as motor. Pains, colicky attacks, hyper-acidity, vomiting, capricious appetite, anorexia, a sense of fullness, eructations, etc., up to a full developed nervous dyspepsia with asthenia, are among the complaints of the patient. Ewald enumerates twenty different troubles, Einhorn even more, though his nomenclature is based mostly upon symptoms, and could therefore be enlarged *ad libitum.* Very often the so-called "nervous heart" accompanies the gastric neurosis to such an extent even as to make it the most prominent point of the complaint. Difficult respiration is also often due to compression of the lungs by a distended stomach. These patients are the unfortunates who go from doctor to doctor, submit themselves to electrical, hydropathic and douching treatment, in fact to everything that the versatile mind of the specialist may suggest.

While surgery has so far been very slow in stepping in, it will, unless I am greatly mistaken, eventually assert itself more and more in such cases. The same process which took place in gynæcology when long standing neuroses were unmasked by the knife, will take place here also; and if I foresee aright, the future specialist of gastric diseases will not be a one-sided internist, but a combination of both, internist and surgeon, exactly as the modern gynecologist.

I will now proceed to point out what diseases, seemingly gastric, may be due to distant causes, and I will confine myself mostly to those having surgical relations, as stated before.

We will begin with the brain. Vomiting is a well known symptom of certain diseases of the brain and meninges, especially in children. It also accompanies injuries, abscesses and tumors of these structures, especially those of the cerebellum. In the latter it may simulate a gastric neurosis, if the nausea and vomiting preponderate much over the more characteristic symptoms, like incoordinate gait, etc. But as a rule there will hardly be much doubt as to the stomach's role. It is different in epilepsy, where the alarm, the initial signal, is given by the stomach. Here the patient will state that a fainty sensation, a painful contraction, and perhaps also vomiting, will constitute the beginning of the attack. I do not doubt that the muscles of the stomach are connected with the gray matter of the brain, just as the muscles of the extremeties are, so that they also have a distinct centre in the so-called motor zone. Up to date no such centre has been verified, the discoveries of some experimenters not yet having been substantiated.

The circuit in these attacks may be presumed to start either at its gastric origin or in that of the brain. In the first instance a certain irritant would, by way of centripetal conduction, excite an abnormally irritable brain-centre to action which, in its turn, would cause a morbid reaction on the dependent musculature in the stomach. Such a mechanism would explain why a counter-irritant, taken just when an attack threatens, may prevent an outbreak. I have had occasion to satisfy myself of this by seeing patients swallow a handful of salt, a popular remedy in some countries. They often abort an attack by this means. This assumption would also explain why dietetic mistakes produce epileptic attacks. The reverse theory that an abnormally acting or diseased brain-centre may first be excited by some unknown cause, and may then, by way of the centrifugal fibres, excite a vicious action of the muscles or nervous structures of the stomach, is equally justifiable. In either case, if the attacks are very frequent, but of a mild type, and not accompanied by loss of consciousness or convulsions, they are liable to be mistaken for purely digestive troubles, so-called "gastric attacks," "spells of indigestion," etc. I hardly doubt that the frequent failures to benefit cases of Jacksonian epilepsy by removing parts of the motor zone, are partially due to a wrong localization from overlooking the stomach as a possible seat of the initial signal. As soon as the proper centre is discovered surgery will claim the right to be given a trial here, just as it has been in instances where the muscles of the extremeties have been thought to give the signal. Whether a centripetal or a centrifugal chain be acting, the circuit would be broken in either instance by the removal of the brain-centre.

We will now turn our attention to the eye. It is a well established fact that gastric neurosis accompanies certain eye troubles, and that it is cured when these disturbances have been cured. As an example muscular insufficiency may be mentioned. It is an equally well ascertained fact that certain disorders of the nose, pharynx, tonsils, palate, etc., which are amenable to the knife, may cause gastric disturbances of grave aspect. A nasal polypus, an elongated uvula, ulcerated tonsils, etc., will either by reflex or by producing toxic substances, which find their way into the stomach, produce these manifestations. I will here cite an interesting case for which I am indebted to Dr. Burg. A woman, fifty years of age, had complained of gastric troubles for many years. Heaviness after eating, a pressure over the stomach, crampy attacks, pyrosis, nausea, occasional vomiting, loss of appetite, etc. Patient was very anaemic, although she had been treated for gastric disease by a number of our most eminent colleagues. Examination failed to reveal anything whatever. By accident the doctor's attention was called to the nose, where he detected a polypus in the left nostril. The removal of the polypus, together with some swollen mucus membrane relieved the patient of all her gastric symptoms at once. This observation corresponds with the statement of Fliess (Die nasale .Reflex neurose, etc., Wiener Klinische Rundshau, 1895), who insists that the anterior third of the left middle cartilage is the place from whence a reflex upon the stomach, emanates and that its removal will cure the neurosis. In addition I would quote Spirk (nasal affections as a factor in chronic gastritis, Philadelphia Polyclinic, 1896), who accuses ozœna of being a source of such neuroses, partially in consequence of swallowed effete substances, and partially by reflex action. Dunn (*New York Medical Journal, 1894*) reports two cases of hyperemesis lasting for years, caused by abnormally enlarged uvulæ; also another case where nasal swelling and deviation of the septum caused the trouble. Swollen papillae of the tongue were the cause of gastric neurosis in still another case.

A richer source, by far, for our trouble is the genital system. In the male, injuries and diseases of the testicles often cause a more or less continuous sickly feeling. But much more frequent are phimosis, and preputial adhesions, which may give rise to all kinds of reflex neurosis, especially in children. I have seen, together with the late Dr. Cupples, a case of suspected gastric cancer get well after the removal of a small urethral stone which had lodged itself in the meatus urethræ and had been there retained several months behind a phimosis.

Diseases of the female genital organs are most intimately connected with gastric neurosis. In fact, there is hardly a female disorder in which the stomach does not participate in one way or the other; mostly though in dislocations of the womb and ovaries, especially of the latter, which permits them to be squeezed and pressed upon. An even more common source are inflammatory and suppurative processes in the generative system. In these cases a reflex action is as much responsible as toxic irritation. The reflex will often be broken at once as soon as the retroflexion or prolapse, is replaced, and the toxic process will cease when the ovarian abscess has been removed. It is hardly necessary to illustrate these statements by cases, as everyone of you will have had experience with them. I wish however to mention two conditions, not exactly surgical, which are highly interesting in this connection.

One is the so-called morning sickness of pregnancy. I believe this is caused by stagnant secretions, due to narrowness of the outlet in an infected cervical canal, for which reason the trouble happens mostly in the primipara. It is a true toxic neurosis and I believe analagous in its mechanism to the before mentioned morphia action on the stomach. It is almost always releived by freely dilating the cervix and then treating the mucous membrane antiseptically so as to check the septic process in the cervix. The failures are generally due to the want of persistance in the treatment and in not giving enough outlet. The dilatation must be thorough, repeated a number of times if necessary, and the mucous membrane thoroughly disinfected.

The second disorder I would mention, is sepsis, resulting from the retention of putrid placental remnants. This process may have gone on for months without having attracted attention to the womb. I have seen several such cases feign the most serious and dangerous gastric diseases.

I will now take up intra-abdominal conditions which are liable to cause gastric disturbances of neurotic nature. They are almost without end. I will only touch upon those which call for surgical interference. They deserve a closer discussion because they are not always as amenable to diagnosis as the ones already mentioned. I will, in conformity with my plan, exclude diseases of the stomach proper, except when they bear a special relation to those under discussion, and I will add that gastric neurosis is mostly complicated with intestinal neurosis.

Aside from degeneration of the nervous plexus located in the walls of the stomach (Auerbach and Meissner) which has been verified in a few instances under the microscope, I believe that there is a disease entirely confined to the nervous centres located in the abdomen, and particularly of the solar plexus, the consequential changes in the depending structures, of course, supervening later. I will never forget a strange case, occurring in a man aged thirty, who was seen by a number of our practitioners. He was absolutely unable to eat. He really had no desire to do so (anorexia) and he became sick from even the smallest quantity of food. It was difficult to understand what kept up his vitality for so many months. He was pitiably emaciated, while mentally he was perfectly normal. He complained of no special trouble of any kind with the exception of one-sided sciatica, which was relieved by one hypodermic injection of antipyrine which produced almost perfect anæsthesia. When he died a post morten was held by Drs. Duke and Braunnagel, his last attendants, who kindly invited me to be present. Nothing abnormal was found except the total disappearance of the musculature of the stomach and bowels which were as transparent as the gut prepared for sausage making. The large abdominal ganglia were unfortunately not looked for. It would have been exceedingly interesting to have had a microscopical examination made of them. It is most likely that they were atrophied, and that the trophic nerves of the stomach and bowels had thereby been set at rest. I saw a similar case, but without a post mortem examination, in the person of a young married German lady. She positively could not eat, and gradually faded away. There was nothing else to be made out in her case.

Next I would call your attention to sub-diaphragmatic abscesses of a more of less obscure nature. They not only cause dyspepsia, vomiting, etc., through toxic influence, but also by direct pressure upon the stomach. I recollect a case seen with Dr. Keller of Marion, where a gentleman suffered for months in this way. He was at once relieve by opening the abscess which fortunately pointed out directly under the ensiform cartilage. A similar case is reported by Koerte (Centrnl bl. fuer Chirurg, 1897). A man complained of severe pains in the stomach with vomiting and fever. A diagnosis of perforating ulcer was made. Upon incision a subdiaphragmatic abscess was reached and drained, and the patient was perfetly restored to health. Fever, dullness upon percussion and a previous history pointing towards gall stones, gastric ulcers, etc., will be of help in the diagnosis. It is true the abscess will often remain unrecognizable if of limited extent.

We will next consider gall-bladder and gall-duct diseases. An acute inflammatory attack of the gall-bladder or a sudden gall-stone trouble will always be accompanied by gastric disturbances. But of greater interest to us is a chronic condition dating back perhaps for years, where the causative trouble is little or not at all evident. It is difficult to find a perfectly satisfactory reason why the gall-bladder, with its appendages should have such an influence upon the stomach. One can easily understand that a stone or a tumor may compress the pylorus, or that an adhesion between the two organs may drag the stomach in one or the other direction, interfering with propulsion or with the peristaltic action; but the gastric neurosis will appear even if all such factors are missing. There must be some toxic influence, no doubt. As to the diagnosis it may be simple. Jaundice, pains over the gall-bladder region, light fæces, etc., and on the other hand absence of proofs of gastric diseases may leave no doubt. But there certainly are cases where nothing but a probatory incision will permit of a correct diagnosis. Especially is this the case in malignant troubles where the stomach, the pancreas, the liver, etc., may participate to such an extent as not to admit of an unquestionable decision as to the starting point, even on the post mortem table. Out of my own practice I can cite a case where a man who was treated for cancer of the stomach by some, and for chronic catarrh by others, was relieved by simply dividing a flat band between the gall-bladder and stomach. I can further cite a case of Dr. Kingsley's, of a lady still living here, who was relieved of a long-standing gastric trouble, which had made her a perfect wreck, by simply breaking up adhesions around the gall-bladder, and only partially at that. An old lady in Dr. Paschall's practice challengened the diagnostic acumen of four of us, all old practitioners. The complaints were always directed to indigestion and gastric trouble. The operation revealed gall stones. Another case was that of Dr. A. C. McDaniel's, whom I assisted in operating for frequent attacks of vomiting and indigestion; but pains over the gall-bladder and occasional jaundice led him to suspect that the latter organ was the seat of the trouble. The gall-bladder was found to be fastened and surrounded by adhesions. These were broken up and the patient made a rapid recovery. Numerous such cases are recorded in literature and they are of the most diversified nature.

The pancreas stands in a somewhat similar relation to the stomach. Here again, already the acute disease shows close connection between the two organs because gastric disturbances are never absent. In chronic cases the gastric symptoms are so prominent that the differential diagnosis is by no means easy. Here also new formations, stone in the duct, etc., may press on the stomach directly, or they may interfere with the flow of secretions or act through toxins indirectly. The differential diagnosis must be based mostly upon the exclusion of gastric disease, unless such signs as sugar in the urine, fatty stool, severe salivation, etc., come to our rescue. Deep seated pains upon pressure may help us out some. Jaundice may be present due to pressure of the pancreas on the gall-ducts. If a tumor can be felt, its position and relation will mostly permit a correct recognition, although even here cases may turn up which will not admit of even a probable diagnosis. As the posterior wall of the stomach lies upon the pancreas with only a double sheet of peritoneum intervening, the differentiation of the seat of the tumor may be impossible. From tumors in the anterior wall of the stomach a pancreatic tumor can be distinguished by bloating up the stomach with gas, when the first tumor will rise and the other become more remote and disappear. A curious symptom, a tetania of the stomach, was seen by Berlitzheimer (Berl. Klin. Woch. 1897) in a case of cysts of the pancreas. According to Holzmann free salivation with rise of temperature is in favor of pancreas disease (Munich Medical Woch. 1894). A case was recently operated on here at the city hospital where total anorexia and pains in the stomach existed with enormous gaseous distension and constant vomiting. But the urine contained sugar and the stools were of a peculiar light chocolate color. Therefore pancreatic trouble was diagnosticated. Operation revealed an abscess in the pancreas which was otherwise atrophic and firmly adherent to the stomach and liver. A peculiar perversed appetite for certain things was seen by me in a case of pancreatic cyst, but I will not say how much of this phenomenon is pathognomonic.

The liver may also directly interfere with the stomach. In a post-mortem operation made this year at the city hospital, we found a pylorus so firmly compressed between the pancreas and liver that nothing could pass through, and we then understood why the patient, who was in the last stages of pulmonary tuberculosis and therefore non-operable, had such constant vomiting and anorexia.

The kidney is another frequent contributor toward gastric neurosis. A sickly feeling results whenever this organ is squeezed, of which fact we can convince ourselves at any time. It is no wonder then that a constant irritation of it, like by a stone, will keep up abnormal gastric sensations. But the floating kidney, above all others is guilty of such an action. Here gastroneurosis is almost a constant symptom though we must not lose sight of the fact that ptosis of the kidney often occurs in conjunction with that of the stomach, as a part of the general dropping (enteroptosis) and threfore a large portion of the suffering may be due to the gastric dislocation. This however, does not come under my present review. The kidney may also compress the ascending part of the duodenum by its wrong position, and thereby interfere with the stomach's execution or the passage of bile downward. The differential diagnosis requires a search for the kidney (mostly the right one) in different positions, especially while standing, when on deep respiration the organ my be felt in front under the arch of the ribs. The urine must be examined as a matter of course. The patient will generally state that he feels much relief when in the recumbent position.

I may add that diseases of the renal capsule have also been found to cause severe gastric distress. A case is reported by my namesake—a Berlin physician—where a tuberculous capsule had caused excessive pains and vomiting. It was successfully removed.

Even the spleen may give rise to gastric neurosis. In a case where an atrophic spleen had become dislocated into the pelvis, great emaciation resulted from inability to take enough food. Constant nausea and painful and fainty sensations in the stomach prevented the patient from keeping her strength. The removal of the organ relieved these symptoms entirely.

I must now direct your attention to a quite frequent trouble which to some extent involves the stomach itself, though not a disease of this organ in a narrower sense. I refer to perigastritis. It is an inflammation of the serosa of the stomach and of the surrounding tissues and organs, leading to adhesions. Those with the gall bladder and pancreas have already been discussed. They may as well form with either lobe of the liver, the diaphragm, the transverse colon, the splenic flexure or with the abdominal parietes. These adhesions may either be flat attachments of two surfaces, mostly the serosa, or be band or thread-like structures. They are due principally to suppurative processes either from diapedesis of germs or percolation of toxic fluids, or to perforative ulcers. The latter occur mostly in the stomach itself, heal and leave nothing but the adhesion. The symptoms are very vague. By excluding real diseases of the stomach, the most reliable sign will be an aggravation of dragging pains in limited places by special movements. The attachment of the stomach to abdominal parietes must be differentiated from a hernia in the linea alba (epigastric hernia) on which point I will dwell hereafter. In either instance a very circumscribed point will be found painful on pressure, and in both cases an incision will be found necessary. I do not doubt that abnormal congenital bands within the region of the gastro-hepatic ligament and extending to the right and left from it may also be causes of distress. The importance of the whole subject justifies the following quotation from a paper by Prof. Mickulisz, the most experienced living surgeon. He says: "We have to differentiate between two kinds of adhesions (1) a loose agglutination which will cause trouble by interfering with the inability of certain portions of the stomach and by dragging. These adhesions are mostly the sequelæ of benign perigastritis. It cannot be doubted that from such adhesions—to which Lauenstein first called attention—very severe gastralgic symptoms may result, similar to those in epigatric hernia. The diagnosis is very difficult, because idiopathic cardialgia may exactly simulate it, and it is possible that the adhesion only excites an already existing neurosis of the stomach. Otherwise I could not find an explanation for the fact that some adhesions—especially after operations—do not lead to such suffering. Nevertheless Von Hacker has proven that very obstinate gastric troubles have been relieved immediately by gastrolysis (freeing of the stomach). I myself (Mickulisz) have only a short time ago operated on a young man whose pyloric portion was dislocated upwards by adhesions. It was turned around its long axis and did not show any evidence of an ulcer. The patient's severe gastric suffering, which had lasted for years, disappeared at once after the gastrolysis.

It is easier to understand the second kind of perigastric adhesions. They form at the site of a perforating ulcer, between the stomach and the overlying abdominal wall. They often form veritable tumors. Mostly, extremely severe pains exist in or around the stomach aggravated by pressure upon it, and by every movement of the patient. Operation is of course indicated." (German Society of Surgery)."

Whoever desires to do this kind of abdominal work must study these conditions most thoroughly so as to avoid mistakes in either direction, i. e. he may mistake unusual ligamentous structures for pathological conditions, or he may overlook really vicious ones. It is also necesssary to know thoroughly the anatomy of the surrounding parts, especially that of Winslow's foramen. Byron Robinson's studies, published in last year's Medical Record are in my opinion the most exhaustive ones to be found, and cannot be too highly appreciated.

It is now in order to inquire what success follows the liberation of the stomach—the gastrolysis. Complete satisfaction is usually obtained after liberating adhesions to the abdominal parietes, and such between the gall bladder and stomach. A number of cures have also been reported after freeing the liver from the stomach, although in some of them the effect was not very thorough. Other disorders will evidently also accompany these adhesions. Much information may be obtained from an article in the *Revue de Chirurgy*, February, 1897, by Tuffier and Marchais. I have obtained a complete recovery in a man upon whom I operated twelve years ago, and who sends me an occasional report now. He had a flat adhesion of the stomach to the middle line, not larger than a nickle, the release of which relieved him of many years of suffering. Another case where the gall bladder was drawn over to the pylorus, died from sepsis, due to a panaritium on the finger of a nurse who handled the instruments. Another case was so complicated that I cannot say whether the bands between the stomach and liver were the real cause of suffering or not. I did a pyloroplasty at the same time, and had to free the omentum. The patient was only partially benefitted. I have already mentioned another case in connection with the gall bladder.

But the stomach is not only handicapped by these bands and adhesions in its immediate neighborhood,—distant ones may also cause trouble. I refer to the adhesions of the omentum, and particularly of its fringes to the abdominal and pelvic walls and organs. I have reopened the abdomen a number of times after operations, when the gastric suffering demanded it, and found the omentum firmly and tightly fastened to the abdominal wall in the line of incision. Once after a vaginal hysterectomy not getting union of the vaginal incision, and the patient unceasingly complaining of a dragging sensation in the stomach, I found an omental fringe adherent to the incision in the vagina. It was resected and great relief obtained. Another time I found the elongated fringes fixed low down in the pelvis, evidently causing gastroptosis by pulling the stomach downward. In another case I found a slender thread of omentum fixed to a point behind the womb, etc. I would strongly advise you to pay attention to these conditions in the diagnosis, and still more so during the operation.

I will next mention tumors pressing upon the anterior wall of the stomach, among which lipomas have acquired some notoriety. Some hold that the small lipoma is the first stage of a hernia. But there is no doubt that such tumors are also found independently. In four cases upon which I operated they were situated directly under the xyphoid processs, and if they were the outgrowths of the omentum, it must have been from the lesser one that they originated. But I had occasion only lately to remove a large omental lipoma, or better termed, lipomotous omentum, which involved the gastrocolic ligament. Its omental structure could only be recognized at its insertion to the colon. In every other respect it was a veritable lobulated lipoma, weighing over one and one-half pounds. It contained but a few small blood vessels. The lady had suffered from all kinds of gastric neurosis and an almost complete anorexia. The most characteristic point, however, was her relative welfare while in the recumbent position, whilst in the erect one she would at once suffer from a dragging sensation on the stomach, with fainty, sickly sensations. All objective symptoms were missing, with the exception of a very tender spot midway between the xyphoid process and the umbilicus in the linea alba. The incision was made over this point, and struck the thickest part of the lipoma, of which a finger-shaped band ran up to the stomach and liver. It afterwards showed a ligamentous string about at its middle, no doubt the round ligament of the liver. This case has not yet progressed far enough to report a final result. I am indebted to Dr. Trion, of Floresville, for this case, and was assisted in the operation by Drs. Burg, Kingsly and my son.

The symptoms are, a pressure on the stomach, especially after meals, and perhaps also a detectable fullness or tumefaction, and absence of real gastric disease. Hyper-acidity is almost always present.

Of my cases, which were all cured by surgical interference, one was seen with Dr. Sykes, in Galveston, another with Dr. Leonards, in New Braunsfels, another with Dr. Shropshire, where an enormously enlarged gall bladder was first removed without sufficient relief, and the last one in a bar-tender here in San Antonio, who had suffered greatly, not only with his stomach, but also from headache, for which latter complication I can find no explanation. He is perfectly free from both troubles now.

The epigastric hernia, and especially that of the linea alba, receives remarkably little attention and discussion in our country, whilst France and Germany have a very rich literature upon it. It is a frequent affection, and in the German clinics you may see from ten to twenty cases operated upon every year. It, more perhaps than any other trouble, is apt to feign all kinds of gastric distress. Von Bergmann reports a case where an army officer was treated for years for a cancer of the stomach. The patient was completely cured by the simple operation for such a hernia. They are ventral herniæ, but mostly of such small size that they are not recognized, unless specially hunted for. A little omental knuckle or a small lump of fat works its way through one of the clefts in the aponeurotic middle line traversed by the blood vessels. By gradually dilating the gap the small lump may drag in more of the omentum, and even portions of the gut. The stomach itself may even enter when the ventral hernia becomes clearly developed. This may also occur at other places in the abdomen; in the semilunar lines for instance.

The diagnosis is made by palpation. A circumscribed spot in the linea alba is then found to be very painful. Perhaps it will also bulge out upon coughing, especially when the patient leans forward. Relief in the recumbent position, together with pains after meals are almost pathognomonic. Litten is of the opinion that a peculiar spurting can very often be felt by placing the finger over such places. Real gastric disease must of course again be excluded. There is no difficulty when the hernia is sufficiently developed and when it differs from an umbilical or other ventral hernia only by its position. I have operated on several such cases with perfect success. My most remarkable case was that of a farmer's boy, twelve years old, who had been a perfect invalid, emaciated and hardly able to walk on account of pain. He is now—two years after the simple operation of the removal of a small piece of fat, and closure of the cleft—a healthy, stout, well nourished boy.

I was not so lucky with a case which is yet under treatment. The patient is a stout Mexican, about twenty years old. He had an extraordinary distension of the large bowels. They stood out in bold relief, worse after eating, when nothing but constant eructations of gas would relieve him for a few minutes. He was afraid to eat, and requested an operation. In the linea alba, about two inches above the umbilicus, upon coughing a distinct elevation of the size of a silver half dollar could be seen bulging out. It was reducible, and clearly a hernia. My assumption was that the adhesions around the hernial ring caught the transverse colon and caused the bloating and gastric symptoms secondarily. The hernia was operated on at the city hospital, by removal of the sac and the stitching of the cleft. It soon became evident, however, that the operation had not fully done its duty, though he is greatly relieved. After repeated search a tumor was detected in the cœcal region, of the size of a hen's egg. It is movable and rounded. The patient has not yet made up his mind to be operated on again, but this will soon become a necessity. An oversight was clearly made at the first examination; and this only emphasizes the old rule, never to be satisfied with anything but methodical thoroughness.

It so happens that I find today an instructive case reported by Chas. Aaron, in the *New York Medical Record*, No. 21. I will quote a few lines from it. "The patient enjoyed good health until four years before he consulted me. While eating his dinner he was suddenly taken with nausea and vomiting. Ever since he has suffered in various ways, and has given such symptoms as to lead his physician to believe he was suffering from stomach disorder. Sometimes he feels comparatively well, and at other times his trouble is aggravated. He complains of pain in the epigastrium. * * * He had lost twenty-eight pounds in four months and was unable to retain food in his stomach. He vomited milk and soup just as he did meat and potatoes, and suffered with constipation. The urine was normal." A small enlargement was felt in the linea alba midway between xyphoid process and the umbilicus. After operation the patient made a full recovery.—In this connection the mere diastasis of the recti muscles in the middle line must be mentioned. As yon all know, it is a frequent consequence of child bearing. But it also occurs in the male. I have a gentleman among my patients who consulted me fifteen years ago for a most annoying belching and a fainty sense of fullness of the stomach after each meal. This caused extreme distress and resisted every kind of medication. An exact examination revealed a cleft of the breadth of a hand in the linea alba, below the umbilicus, no doubt a congenital defect. The wearing of an abdominal supporter relieves him sufficiently to make an operation unnecessary.

As to other herniæ, it is almost an offense to you to say much as to their effect upon digestion and upon the stomach's conduct, as the havoc done by them is one of the best established facts in surgery. It is different with the obscure, non-detectable herniæ; these should be ever kept in mind. In acute primary cases we will also find nausea, vomiting, etc., but the urgency of the case and the apparent intestinal complication will mostly lead to their recognition at once. It is different in chronic cases, where the bowel is frequently caught, not exactly strangulated, but only compressed and squeezed; or where it is permanently held fixed so as to be interfered with in its peristaltic motions, without being entirely prevented from performing its functions. We ought, therefore, to examine the hernial openings very thoroughly in every suspicious case. Unfortunately the hernia may be an internal one, say intraperitoneal and non-palpable. Of these I will first mention the diaphragmatic hernia, which is not as rare as commonly supposed. Thomas has collected as many us 290 cases from literature. Leichtenstern makes a distinction between a true hernia, mostly acquired, where a sac is formed by the peritoneum or pleura covering the viscera and slipping through a slit of the diaphragm into the thoracic cavity, and the false congenital hernia, where an ectopia of the abdominal organs exists, so that they move about freely in the pleural cavity. The hernia occurs mostly in the left side. It is not recognized until incarceration occurs, and even then only one case has been correctly interpreted, so far as I am aware. (Abel.) I wish, however, to call particular attention to cases which never become urgent, but which may, by frequent sudden pains, or a constant aching in the left side, accompanied by gastric unrest, nausea, etc., be recognized after other disorders have been excluded. Such cases are not infrequent, and I remember many a case where I had reason to think of them, but I have never had an opportunity to operate for them yet.

Internal hernia also occurs in Winslow's foramen, and in the fossa duedeno-jejunalis, which is normally a very small pocket, but which was once found to contain the whole small intestine. Such herniæ have been described as many as sixty-four times (Jonesco). They were never diagnosticated, though. Herniæ have also been found in peritoneal pockets connected with the caecum, and between it and the appendix. They may all exist without the gut becoming seriously constricted. Constipation, intestinal distension, cramps, etc., will be the most frequent symptoms; gastric neurosis will always be present. They are recognized as a rule on the post-mortem table. Making a suspect turn and stand in different positions and finding some decidedly painful, may throw a dim light on the matter.

Not long I ago operated on a German woman aet 25, whose case is worthy of a report. For many years she had been an object of pity. Loss of appetite, nausea, occasional vomiting, and constant pain in the bowels and stomach had reduced her to a perfect skeleton. Upon examination, the appendical region was markedly tender, and a chronic appendicitis was duly diagnosticated and operated for. A median incision was made, as is my custom now in all chronic forms of appendicitis. The small bowel had to be lifted out, and here we found a small portion, about one inch in length, squeezed together as with a forceps; of a pale appearance, which, after liberating, gradually recovered its color and size. Proceeding, we found no appendicitis, but a pocket formed by a double fold of an appendical mesentery, having the caecum for its base. It appeared to us that the compressed gut had been caught in this pocket and had been made partially impermeable. The operation was quickly done without a hitch, but the patient died four days later from paralysis of the bowel, no passage being obtainable in spite of a search for the obstruction after reopening the abdomen. Drs. Paschall, Burg, McDaniel and F. Hadra, were kind enough to assist me.

As an example of an unrecognized femoral hernia, a case now under observation may serve. A lady about 50 years of age, from abroad, was seen by me about two weeks ago, during a gastric attack, which presented most distressing features. The pulse was small, quick and irregular; she gasped for breath and held the stomach with both hands. She had these attacks, I was told, off and on for six years. I was also told that she had all kinds of diseases, pulmonary tuberculosis, gastric catarrh, a weak heart, etc. On account of the exquisite tenderness over the gastric region an examination was impossible. A hypodermic injection relieved her sufficiently, and nothing further was done then because her relatives maintained that she always recovered after one of these attacks. On my next visit I found her almost well, but very much exhausted. I also learned that at times she could walk with perfect ease, at other times, though, only with great pains in her left pelvic half. I was told that she also suffered from uterine prolapse. On examination I found no evidence of real gastric disease, heart disease, or abnormal genital organs. But while examining the hernial openings, a little lump was felt in the left femoral canal, evidently either a swollen gland or a knuckle of fat—the frequent concomitant, perhaps the cause (as Roser insisted) of hernia. The case was no longer a mystery to me. I may add that a microscopical examination of the sputum failed to detect bacilli. The lady has not yet consented to an operation.

We will now proceed to a consideration of chronic partial obstructions in the bowels, which are an ample source of intestinal and gastric neurosis. They are characterized by a great loss of appetite, irregular and mostly difficult defecation. Those in the duodenum below the papilla lead to the passage of bile into the stomach, and consequently to bilious vomiting. The obstructions are caused by shrinkage after cicatrization of ulcers of various nature (strictures); by foreign bodies (gall-stones, etc.); by the twisting of the bowel around its long axis; by intussusception; by outside causes, like pressure of tumors or abscesses, or by being dragged by adhesions to one side; by the enwrapping of adhesive bands, or of Meckel's diverticulum; in short, by a great number of causes. A strict diagnosis is often impossible. Localization of pain at a certain spot of the abdomen; the presence of a tumefaction; still more, the constant existence of a bloated condition at a given point, together with the exclusion of other possibilities, are the main guides. As a rule, only surgical interference will offer relief, the exact diagnosis must be left to direct inspection; but it is necessary to have the whole list of disorders in mind so as not to overlook anything, which may easily be done even by an expert, because these chronic forms of which we speak are mostly not as plain as acute cases of constriction.

It is here more than anywhere else necessary to be methodical. In my opinion, the bowels should always be examined from end to end, so the whole omentum, the stomach, the ovaries, etc. I know from experience how easily the very cause of the trouble may otherwise escape our attention. It would be of value to work out certain rules as to the order and the line of these manipulations, and to practice them on the cadaver, if possible, while at school. I will omit citing cases, as the field is too extensive, and because every one of you is, no doubt, acquainted with these occurrences.

The presence of an abdominal abscess will by toxicity exert a similar influence, and I have never seen a more aggravated form of gastric distress than I saw in a Jewish widow upon whom I operated in Galveston with Dr. Fly, for an abscess due to multiple perforation of the ilium, which must have occurred many years before. The perforation had been walled off by other loops of the intestine. After resecting about six inches of the gut the burning of the stomach, and the reching which had existed previously to a moderate extent, became so intense that the patient screamed for hours. Nothing would give her relief. However, after some weeks she recovered entirely, and is now perfectly well.

As to tumors, I am still less prepared to explain their influence upon the stomach, pressure excluded. Even tumors of the large intestine may have such disastrous effects upon this organ. Two years ago I made a laparotomy upon a German lady, 28 years old, who was rapidly wasting away from mere inability to eat and assimilate. A constant nausea and fainty sensation around the stomach was all that could be made out. During the operation I found some adhesions around the stomach, and by breaking them up I hoped to have cured her. But the relief was only slight and temporary. Some months later I detected a swelling in the left abdominal side, which had the appearance of being located directly under the skin. As she was then feverish, and as the swelling was seemingly fluctuating, I took it to be an abscess, due perhaps to some infection from the first operation. Dr. Burg assisted me when I tried to lance the abscess. Not getting any pus, however, I made a small incision, when to our amazement we discovered that the swelling was a tumor in the wall of the descending colon. The wound was enlarged and the gut withdrawn. A soft fibro-sarcoma of the size of a small fist was found in its wall, involving about three-fourths of the gut's circumference. To save the emaciated patient the risk of still another operation the gut was resected with the tumor at once, and the cut ends united. All this was done with little difficulty and good speed, but the patient died in thirty-six hours from exhaustion.

Chronic peritonitis, especially tubercular, is a frequent cause of the most persistent and intense gastric suffering. Inability to take food or to assimilate it, is always present. It would be superfluous to go into its symptomatology at this place.

There is one particular form of chronic intestinal inflammation which I deem of such importance that I will present the subject to you more exhaustively on some future occasion. It is chronic appendicitis of a hidden character, which I think would properly be termed appendicitis occulta. Much is and has been written on appendicitis; still, in my opinion, the subject is by no means exhausted. Yet, at this place I only wish to give my views on a chronic form which is so obscure and so quiescent that it is mostly overlooked. I have, since my attention has been called to it, had occasion to study this condition somewhat intimately, and I can state that a great number of seemingly healthy people have a chronic inflammation of the appendix, as proven by pains upon pressure over McBurney's point, and verified by frequent examinations. But in cases of chronic gastric suffering, which is mostly termed gastric catarrh by slip-shod, quack diagnosticians, I have often found this spot to be so painful that patients who were formerly unable to locate the pains, could, after their attention was once called to it, distinctly make it out themselves. I have operated on a few such cases with perfect success, and among these were several who had been operated upon before for various other troubles, mostly ovarian diseases, proving the connection between appendix and uterine appendages, as lately emphasized by several authors. I might mention also the case of a robust German farmer, who had for years been treated for gastric catarrh. He complained of a constant hyper-acidity and a belching, which would give him no rest. He had not the slightest intimation of any bowel trouble. He was completely cured by the removal of an appendix which had become adherent in its whole length to the caecum, and showed an injected and swollen mucous membrane. Such appendices may outwardly look perfectly healthy, though they prove to be abnormally stiff on closer examination. I would like to draw your attention to such cases. They are characterized by every kind of intestinal or gastric neurosis, and are diagnosed by finding the so-called McBurney's point painful, upon different examinations. There is almost an invariable slight evening rise of temperature. More decidedly developed chronic and intermittent cases are easily made out. They cause sufficient distress to make their detection the rule. But for this particular form one has to hunt and to search.

In conclusion I will mention rectal troubles, especially hemorrhoids, usually accompanied by habitual constipation as factors of gastric neurosis. Fistulæ, ulcers, etc., have, as a rule, the same effect. It is well to think of tapeworm as a possible cause in our country where it is so frequent, not to be occasionally outdone by the tapeworm quack.

After having thus enumerated the great number of disorders which may be the causes of one or the other form of alimentary neurosis, you will naturally ask: whether it is probable that a correct selection can be made between the many possibilities? I can answer affirmatively—in most cases—and that if it cannot be done, an abdominal section must be depended upon to clear up the diagnosis, if the suffering is so great as to demand relief. The cases under discussion are chronic cases, and admit of thorough and oft-repeated examinations.

I will now endeavor to outline an examination for the general practitioner, without going into the minutiæ which the specialist claims to be in possession of. I believe that my sketch will suffice in most cases to arrive at a diagnosis positive enough for a working basis. Of course every physician will in the course of time get used to his own methods, and it will matter little how much they differ in details as long as they are methodical and exhaustive. But for one who is not constantly doing such work, a certain outline is to be recommended, so that he will not accidentally lose sight of some important point. I may add that the following plan does not pretend to be perfect; it is impossible to cover every possibility in practice. But even if I were able to give a reasonably complete survey, I would not do so on this occasion, because it would require a whole book to do the subject full justice. Imperfect as it is, I hope nevertheless to be able to benefit some one of lesser age and experience than mine.

A patient seen for the first time at his house or in the office should be carefully inspected as to his general appearance. We may at once discover evidences of inanition, or at least underfeeding. If to this is added a peculiar, sallow, dry skin (the combined result of inanition and toxæmia) we will at once think of cancer, though we must not be too sure about this. If the patient is over 40 years of age, this will be in favor of our surmise, though it should not be forgotten that people as young as 20 are not proof against this dreaded enemy. If, on the other hand, the patient shows no evidence of under-nutrition, we will have reason to think of a curable disease. In a young woman with a bleached, chlorotic appearance, gastric ulcer will suggest itself. If there is jaundice the possibility of gall bladder or pancreatic disease will impress itself upon our minds.

Finished with these observations we begin to put a few general questions, so as to ascertain the seat of the patient's complaint, after which we enter into the previous history very minutely. The age of the patient, as already stated, is of great importance. The duration of the trouble comes next. A longer period than two years would militate against cancer, though here again exceptions are possible, because cancer may supervene upon a long-standing round ulcer. The longer the disease has lasted, and the less the patient has become reduced, the more does neurosis become possible. Heredity, in my opinion, plays but a very small role. His habits also may direct our thoughts. Hasty eating produces indigestion. Standing or leaning against a desk for hours at a time may cause adhesion or indurations from pressure on the stomach. The occupation must also be taken into consideration—for instance, the handling of lead preparations (lead colics.) The continuity of the morbid symptoms is of great moment. If present it will argue in favor of organic diseases, while neurosis will mostly display frequent intervals of perfect wellbeing. We then inquire into the dependence of the trouble upon eating, and how long thereafter the symptoms set in. This latter point will be of interest only if the phenomenon is constant as to time. If the pain sets in immediately after the commencement of the meal, it will speak in favor of ulcer, cancer or stenosis; if later, it will, according to location, be construed as indicating an impediment in some part of the intestines; and if coupled with a bloated condition of the stomach, lasting during stomach digestion, it will make probable the existence of something pressing or dragging on the stomach from the outside. We must not accord too much diagnostic significance to this sign, though. Hematemesis and bloody stools are already well known as signs of cancer and ulcer. Not to be forgotten are diseases of the liver where hæmatemesis is frequent. Constant vomiting of bile will indicate stenosis of the duodenum. Vomiting must be inquired into, whether right after eating, as in cancer, or in stenosis of cardia, or late and independently, which is more in favor of neurosis. Heartburn, eructations, unrest of stomach, pains and loss of appetite, while of minor importance, on account of their frequent accompaniment in other diseases, should not be overlooked. The position, though, in which the pains are greatest—

standing or lying or leaning forward—is of moment. Bending, for instance, causes exacerbations in adhesions and epigastric hernia. Sometimes the pains will only set in in certain positions. From all this inquiry we will perhaps have already learned enough to answer the question: "Is it one of the organic diseases of the stomach proper, or is it the more indefinite nervous disturbance, the neurosis?"

We must exclude the former before we have a right to assume the latter, and only chronic progressive catarrh upon which I will say a few words later, will rather have to be eliminated in the reverse manner. When the differential diagnosis has been narrowed down to a selection between these two, we will rather have to exclude the neurosis in order to assume the catarrh by which latter term I do not mean the acute form or a passing irritation of the mucous membrame, but a genuine chronic progressive degeneration of the mucosa and especially of the glandular apparatus, which is a formidable and fortunately much rarer disease than commonly thought.

The physical examination of the patient will now engage our attention. This is done in the recumbent position with the whole abdomen fully in view. After looking for some visable abnormalities, protrusions (hernia, tumors), peristaltic movements, etc., we proceed with palpation and percussion. With one or both hands we impress the sides and middle lines for the detection of deeper seated tumors and for resistance of the muscles, the two sides being carefully compared. A quick and firm muscular contraction makes an underlying trouble probable. The tumors we look for mostly are located to the right of the middle line, immediately, or from one or three inches below the margin of the ribs. They may possibly be cancerous or fibrous tumors of the pylorus, more or less moveable, and mostly so to the left, where they may be found at first. They are usually small, not larger than a walnut. Higher up and seemingly connected with the liver we would expect gall-bladder tumors or distensions. They may be of large size and are sometimes covered with tongue-like processes of the liver. More towards the middle line or on the side of it, tumors of the pancreas are sometimes accessible to palpapation. Sometimes they appear to be located almost superficially though they need not be large cysts which would naturally grow in an upward direction and mostly to the left side. Then there may be tumors in the stomach itself. The location and consideration of other points already found or to be further elicited may clear up the diagnosis at once. Thus jaundice will be in favor of gall-bladder or pancreatic tumor. (The urine, stools, etc., will be discussed later.) Dislocated or enlarged kidneys and spleens must be searched for of course. We then examine the coecal region for appendical tumors more closely.

After this more or less gross examination a methodical pressure with one or two fingers should be exerted over every spot of the abdomen. This is of paramount importance, and as it is often the only way to come to a conclusion, it should never be neglected. The middle line should especially be followed inch for inch in its whole length to elicit either undue resistance or yielding, or tenderness or pain. The same procedure should be carried out in the hypochondriac regions. By these painstaking means we will perhaps be enabled to discover or think of proofs for the existence of fatty tumors, of adhesions and before all of epigastric and umbilical herinæ. We should now extend the manipulations to the lower half of the abdomen, especially over the coecal region, where to our surprise exquisite tenderness may sometimes be elicited over a limited spot. This would suggest appendicitis. The left side on the same level may disclose the same symptoms and even in appendicitis we will occasionally find these signs on the left side, either because the appendix is located there or because there is irritation of loop of the next small intestine. Deep palpation is next made in the pelvis to search for female disorders. The bladder must also not be forgotten, and last but not least the inguinal and femoral rings must be examined for small herniæ, the patient being made to cough. Whenever palpation discovers anything suspicious percussion should be added, superficial as well as deep. Through this agency we may detect a dull spot indicating a tumor or a swelling or the distension of a certain portion of the bowel. This latter would be in favor of an impediment with stagnation above it. We now go back and define the out lines of the stomach by percussion. If it is empty it often falls backs upon the vertebral column and is therefore sometimes not amenable to percussion under the margin of the ribs, or the tympanitic sound is continuous with that of the bowel making it very difficult to reach a correct conclusion. Its contour to the left and upwards will, however, almost always be accessible to percussion and mostly allow a correct estimate. If the size of the stomach is of special importance for diagnosis, we let the patient swallow a solution of from one-half to one drachm of tartaric acid, followed directly by a solution of 1 drachm of bicarbonate of soda. The carbonic acid gas so generated will generally bring the stomach into view or at least distend it sufficiently to be outlined by percussion. We may now be able to recognize dilatation or gastroptosis (in which case the lesser curvature will appear below the xiphoid process—otherwise it is not visible). We may also, after the distension, feel a pyloric tumor or one in the anterior wall, much better. Or a tumor felt before will disappear, proving its position to be in the posterior wall or behind it. If indications demand it, a bi-manual vagino-pelvic examination is now added. Where all of these procedures have failed to clear up the case, we should make the patient twist to the right and left in order to discover where he feels the most pronounced distress. This may lead to the recognition of adhesions. A dislocated spleen and kidney will also be more easily recognized by this means. In leaning forward or backward in the standing position even more points may be elicited. An epigastric hernia, for example, may be more easily made out in a stooping position. It will at times also be appropriate to examine the patient for locomotor ataxia, in which disease gastric attacks are well known occurrences.

It now remains to add that in questionable cases for the purpose of excluding certain diseases of the stomach, or to confirm the participation of the gall bladder or pancreas, we must examine chemically the gastric juices and the urine. The stools, too, must be closely observed, for all of which we take our time. The methods for making gastric chemical examinations are much less cumbersome and difficult than generally supposed. They are just as easily within the reach of the general practitioner as urinary examinations are. They are described in the text books more fully than I can give them to you. Suffice it to say that the diagnosis of cancer will be greatly strengthened if we find an absence of free hydrochloric acid, and the presence of an undue amount of lactic acid. I further believe that hyper-acidity will mostly be present in neurosis from nervous over-stimulation. A prolonged retention of the contents of the stomach—proven by the use of the stomach tube seven hours after a meal—will show evidence of muscular weakness, stenosis, etc. This condition is rarely present in pure neurosis. But as this latter may lead to inertia of the stomach, or be a concomitant of it, this test may sometimes leave the the diagnosis doubtful. Bile pigments in the urine, and clay-colored stools will prove impediments in the bile ducts. Sugar in the urine and fat in the stools will suggest pancreatic trouble. Casts in the urine and an abnormal quantity of crystals will direct our attention to kidney disease. Indican in the urine is a point in favor of stenosis of the small intestine, etc.

I will bring my long article to an end by repeating that the term, *catarrh*, is the most abused one of all names for stomach diseases. It is the ready conclusion of the superficial practitioner, and the most common and convenient method for disposing of obscure troubles. We are only justified in making such a diagnosis when every other gastric disease, and when the neurosis, can be excluded. This will be done when a careful examination does not reveal anything that could be construed as a source of the neurosis. Furthermore, we will hardly expect to find a capricious alternation of good and bad days or weeks in a progressive degeneration of such important parts of the stomach as the mucous membrane and the glandular structures. Tissues once destroyed are not likely to act anew occasionally. For the same reason, it is my opinion that the presence of hypersecretion of gastric juices militates against the assumption of chronic catarrh, while over-stimulation in neurosis makes its existence but plausible; and lastly, a constant progressive tissue waste, with permanently diminished kidney secretion as to quantity and quality; is surely rather in concert with catarrh than with neurosis.

